# Development of an antibody-dependent cellular cytotoxicity reporter assay for measuring anti-Middle East Respiratory Syndrome antibody bioactivity

**DOI:** 10.1038/s41598-020-73960-x

**Published:** 2020-10-06

**Authors:** Junxia Cao, Lan Wang, Chuanfei Yu, Kaiqin Wang, Wenbo Wang, Jinghua Yan, Yan Li, Yalan Yang, Xiaomin Wang, Junzhi Wang

**Affiliations:** 1 Key Laboratory of the Ministry of Health for Research on Quality and Standardization of Biotech Products, National Institutes for Food and Drug Control, No. 31, Huotuo Road, Biomedical Base, Daxing District, Beijing, 102629 China; 2 Department of Physiology and Pathopysiology, Capital Medical University, Youanmen, Fengtai District, Beijing, 100069 China; 3 CAS Key Laboratory of Pathogenic Microbiology and Immunology, Institute of Microbiology, Collaborative Innovation Center for Diagnosis and Treatment of Infectious Diseases, Chinese Academy of Sciences, Beijing, 100101 China

**Keywords:** Biological techniques, Drug discovery

## Abstract

Middle East Respiratory Syndrome coronavirus (MERS-CoV) is a highly virulent pathogen that causes Middle East Respiratory Syndrome (MERS). Anti-MERS-CoV antibodies play an integral role in the prevention and treatment against MERS-CoV infections. Bioactivity is a key quality attribute of therapeutic antibodies, and high accuracy and precision are required. The major methods for evaluating the antiviral effect of antiviral antibodies include neutralization assays using live viruses or pseudoviruses are highly variable. Recent studies have demonstrated that the antibody-dependent cellular cytotoxicity (ADCC) activity of antiviral antibodies is more consistent with the virus clearance effect in vivo than neutralization activity. However, no reports evaluating the ADCC activity of anti-MERS antibodies have been published to date. Here, we describe the development of a robust and reliable cell-based reporter gene assay for the determination of ADCC activity of anti-MERS antibodies using 293T/MERS cells stably expressing the spike protein of MERS-CoV (MERS-S) as target cells and the engineered Jurkat/NFAT-luc/FcγRIIIa stably expressing FcγRIIIA and NFAT reporter gene as effector cells. According to the ICH-Q2 analytical method guidelines, we carefully optimized the experimental conditions and assessed the performance of our assay. In addition, we found that the ADCC activity of afucosylated anti-MERS antibodies is higher than their fucosylated counterparts. The establishment of this ADCC determination system provides a novel method for evaluating the bioactivity of anti-MERS antibodies and improving ADCC activity through modification of N-glycosylation of the Fc segment.

## Introduction

Over the past two decades, three coronaviruses have appeared in the world and their outbreaks have caused considerable global health panic. Severe Acute Respiratory Syndrome Coronavirus (SARS-CoV) was appeared in 2003 and lead to a case fatality rate of 10%^1^. Middle East Respiratory Syndrome Coronavirus (MERS-CoV) emerged in 2012 is still circulating and the case fatality rate is much higher (around 37%)^2^. Recently, a new coronavirus named COVID-19 is spreading overall the world. Despite the lower case fatality rate, COVID-19 has infected much more people and resulted in more deaths than SARS and MERS combined^3^. The threat of global pandemic of diseases caused by these coronaviruses has raised global concerns. However, the treatment measures for these diseases caused by coronaviruses are symptomatic, because there are no specific vaccines or coronavirus antivirals. MERS-CoV has the highest mortality rate among these coronaviruses, and current treatment options for MERS-CoV infections have been adapted from treatments for SARS outbreaks in 2003 and/or H1N1 influenza outbreaks in 2009^4,5^. Several vaccines and drugs for the prevention or treatment of MERS-CoV infection are currently under investigation, but none have been approved for clinical use^5–9^. Among these vaccines and drugs, passive immunotherapy in vivo using neutralizing monoclonal antibodies (mAbs) has been reported to be effective in the prophylaxis and treatment of MERS-CoV infections^9^. Johnson et al. demonstrated that 3B11-N, a mAb against MERS-CoV, reduced pulmonary pathology in rhesus monkeys infected with MERS-CoV^10^.

The genome of the MERS-CoV is a single, positive-stranded RNA that encodes at least 10 open reading frames (ORFs) which are translated into several viral structural proteins, including spike (S), envelope (E), membrane (M), nucleocapsid (N), and accessory proteins^11^. The highly glycosylated spike (S) protein mediates receptor binding and membrane fusion and is the main determinant of viral entry and infections^12^. The S protein consists of two subunits: the S1 subunit mediating the attachment of viral particles onto the cell surface, and the S2 subunit mediating fusion of the virus with the cell membrane of the host^13^. The receptor-binding domain (RBD) in the S1 subunit is responsible for virus attachment to dipeptidyl peptidase 4 (DPP4, also known as CD26), which acts as the cellular receptor for the MERS-CoV^12,14,15^. The RBD region is the major target for the development of MERS-CoV neutralizing antibodies. Currently, nearly 20 different neutralizing monoclonal antibodies have been identified and characterized by several research groups with various approaches^9,16–26^.

Measurement of the bioactivity of therapeutic mAbs is critical in antibody development and quality control. The major method for evaluating the anti-virus effect of the antibody drugs includes neutralization assays using live viruses^19,27^ or pseudoviruses^17,24^, and animal infection protection experiments^22,24^. Animal experiments are generally expensive and complicated to operate and their use in quality control and routine release of therapeutic drugs are hindered. The live virus neutralization assay requires biosafety level 3 (BSL-3) facilities and skilled operators. Pseudoviruses with limited infection and replication ability can overcome the safety difficulties of live viruses, and the neutralization assays using pseudoviruses showed highly consistent results with that of the live virus-based neutralization assay^28^. Traditionally, the neutralization activity mediated by the interaction of the antibody Fab segment with the virus antigen was considered as the most important function in the virus clearance. However, recent studies have demonstrated that the contribution of non-neutralization activity mediated by the Fc segment of antiviral antibodies has often been overlooked.

Antibody-dependent cellular cytotoxicity (ADCC) is triggered by binding of the Fc segment of antibodies to the Fc receptors (FcRs) on effector cells such as natural killer (NK) cells, γδ-T cells, macrophages, and neutrophils, thereby inducing these effector cells to release cytotoxic substances such as perforin and granzymes^29^, resulting in the elimination of infected cells and inhibition of viral spread^30^. It has been reported that the ADCC activity of antiviral antibodies plays an important role in the clearance of viruses such as Ebola^31^, Marburg^32^, HIV^33,34^, Lassa virus (LASV)^35^, and influenza virus^36^. Notably, there are reports that the ability of anti-Ebola antibodies to clear the virus in vivo is attributable to ADCC activity, regardless of whether they have high neutralization activity^37,38^. Hiatt et al. explored the contribution of effector function to palivizumab efficacy and found that enhancement of ADCC activity by removal of the fucose glycan on the antibody significantly enhanced the effectiveness of palivizumab, which is approved for preventing RSV infections in at-risk neonates^39^. Thus, the ADCC activity is more consistent with the ability of the antibody to clear the virus in vivo than the neutralization activity. However, to date, no reports evaluating the ADCC activity of anti-MERS antibody have been published.

In this study, we report the development of a robust and reliable cell-based reporter gene assay (RGA) for measuring the ADCC activity of human anti-MRES-spike antibodies. ADCC activity was measured using target 293T/MERS cells that stably express MERS spike protein on their surface and effector Jurkat/NFAT-luc/FcγRIIIa cells that stably express FcγRIIIa and luciferase reporter genes driven by NFAT response elements. This assay is not reliant on live viruses or pseudoviruses and thus is safe and convenient to operate, and shows good performance characteristics, including high specificity, stability, accuracy, precision, and robustness. Establishment of this ADCC determination system can provide a novel evaluation method for the bioactivity of anti-MERS antibodies.

## Materials and methods

### Antibodies and plasmids

Anti-MERS antibodies of murine 2E6 (m2E6), humanized 2E6 (h2E6), afucosylated h2E6 (AF-h2E6), and optimized coding sequences of the MERS-CoV spike protein (MERS-CoV S) were gifts from Professor Yan of the Institute of Microbiology, Chinese Academy of Sciences, and the details were described as previous^20^. The PNGase F-treated AF-h2E6 (PN-h2E6) was derived from the AF-h2E6 antibody treated with PNGase F (New England Biolabs) overnight at 37 °C. The recombinant plasmid (pcDNA4-MERS-S) containing the optimized sequence of the MERS spike protein.

### N-linked glycosylation analysis

The N-glycosidic-bound oligosaccharides were released by PNGase F and labeled with 2-AB (Glyko Signal 2-AB Labeling Kit, ProZyme), followed by removel of excess 2-AB. Labeled N-glycan chains were analyzed by HILIC-UHPLC using a Waters BEH Glycan Separation Technology column (2.1*100 mm, 1.7 mm) on a Waters ACQUITY UPLC system. A 60-min acetonitrile gradient was applied and fluorescence signals were detected at 420 nm (excitation at 330 nm).

### Preparation of the MERS pseudovirus and the pseudovirus neutralization assay

MERS pseudovirus preparation and the pseudovirus neutralization assay were performed as previously described^20^. Briefly, 293T cells were co-transfected with Env-defective and luciferase-expressing HIV-1 backbone plasmid pNL43-Luci and recombinant plasmid pcDNA4-MERS-S. The supernatant containing the MERS-CoV pseudovirus (p MERS-S) was harvested 48 h after transfection, and the 50% tissue culture infectious dose (TCID_50_) of the p MERS-S was determined by infection of Huh7 cells. After incubating 100 TCID_50_/well pseudoviruses with fourfold serially diluted antibodies (0.76 ng/mL to 50 μg/mL) for 30 min at 37 °C, the mixtures were employed in infecting the Huh7 cells for another 48 h at 37 °C. Luciferase activity was measured using Bright-Glo Luciferase Assay System (Promega).

### FcγRIIIa binding analysis

The affinity of anti-MERS CoV antibodies to the human FcγRIIIa (CD16a) was determined using the Octet Red96 system (ForteBio). The biotinylated recombinant human CD16a protein (Sino Biological Inc) was equilibrated in kinetics buffer (phosphate-buffered saline, 0.1% bovine serum albumin, 0.02% Tween-20) at a concentration of 5 μg/mL onto the streptavidin sensors (ForteBio). The anti-MERS antibodies were equilibrated with kinetics buffer at two-fold serial dilution concentrations from 31.3 nM to 4000 nM. The sensors were regenerated by dipping in the regeneration buffer (150 mM NaCl, 300 mM sodium citrate, pH 3.5) and wash solution alternately to remove the antibodies but not the CD16a loaded on the sensors. The Octet assay protocol was performed as follows: baseline (kinetic buffer) for 60 s, loading (rhCD16a proteins) for 150 s, baseline 2 (kinetic buffer) for 60 s, association (anti-MERS antibodies) for 60 s, disassociation (kinetic buffer) for 60 s, regeneration (regeneration buffer) for 5 s and wash (kinetic buffer) for 5 s alternately for three times, then the next recycle was started from the association process at a higher concentration of the same sample. The data of the samples were subtracted from the value of the control sensor and were analyzed using the analysis software provided in the Octet Red96 system.

### Generation of the 293T/MERS cell line

The 293T cells (ATCC, CRL-3216) were transfected with the pcDNA4-MERS-S expression vector using the Lipofectamine transfection system (Invitrogen), followed by zeocin selection (500 μg/mL) 24 h post-transfection. After recovery, the stable pool was subcloned by limited dilution, and the clones with high expression of MERS-S were identified by flow cytometry. For the flow cytometry assay, the cells were incubated with h2E6 anti-MERS antibody against the cell-surface antigen for 30 min at 4 °C and the unconjugated antibodies were washed out with FACS washing buffer (2% FBS in PBS), followed by another 30 min incubation with APC-conjugated anti-human IgG Fc antibody (Biolegend) and washing procedure. Isotype antibodies were included, substituting the anti-MERS antibody as negative controls. The luminescence intensity of the stained cells was analyzed by flow cytometry (FACS Calibur, BD Biosciences). The clones highly expressed the MERS antigen, which were identified by flow cytometry, were further selected by the ADCC reporter gene assay (RGA). The selected 293T/MERS cell line was cultured in high glucose DMEM medium with 10% fetal bovine serum and 500 μg/mL zeocin (Gibco).

### ADCC reporter assay using Jurkat/NFAT-luc/FcγRIII***a***

To assess the antibody-dependent cytotoxicity of anti-MERS antibodies, an FcγRIIIa reporter assay was performed and optimized. Jurkat/NFAT-luc/FcγRIIIa effector cells were developed as previously described^40,41^. The target 293T/MERS cells and the effector Jurkat/NFAT-luc/FcγRIIIa cells were counted and diluted in the dilution medium (RPMI 1640 with no phenol red and with 2% low IgG FBS) and were seeded into white 96-well plates (2.5 × 10^4^ 293T/MERS cells/well in 25 μL of dilution medium and 1 × 10^5^ Jurkat/NFAT-luc/FcγRIIIa cells/well in 25 μL of dilution medium, and the ratio of the effector to the target cell was 4:1). Anti-MRES antibodies were serially diluted in a 1:2.5 ratio with dilution medium at an initial concentration of 2 μg/mL, and 50 μL of the serial antibody diluents were added into the 96-well plates. Cultures were incubated at 37 °C with 5% CO_2_ for 16 h. By adding of Bright-Glo Luciferase Assay reagent (Promega) and measuring relative luciferase units (RLU) with a plate reader (SpectraMax), the ADCC activity of anti-MERS antibodies can be determined. In this study, the ADCC reporter gene assays that have not been described specifically refer to this method.

### ADCC reporter assay using NK92/NFAT-luc/CD16a

For method comparison, NK92/NFAT-luc/CD16a cells were used to replace Jurkat/NFAT-luc/FcγRIIIa cells as effector cells. The 293T/MERS cells and the effector cells NK92/NFAT-luc/CD16a were counted and mixed at the proportion of 1:2 (5 × 10^5^ 293T/MERS cells/mL and 1 × 10^6^ NK92/NFAT-luc/CD16a cells/mL), and the mixtures were seeded into white 96-well plates in 50 μL of assay medium dilution medium. Anti-MRES antibodies were serially diluted with dilution medium with a 1:5 ratio at the initial concentration of 100 μg/mL, and 50μL of the serial antibody diluents were added into the 96-well plates. The cultures were incubated at 37 °C with 5% CO_2_ for 16 h. The luciferase activity was assessed by the addition of Bright-Glo Luciferase Assay reagent.

### Accuracy and linearity

AF-h2E6 anti-MERS antibody was diluted to 1, 1.5, 2, 2.5, and 3 μg/mL in dilution medium with the 2 μg/mL concentration as the reference standard. The expected bioactivities of the serial dilutions were 50%, 75%, 100%, 125%, and 150% respectively. Linear analysis was performed using the measured versus the expected potency of each concentration point. The ratios of the measured potency to the expected potency were used to determine the accuracy of the method. All of the samples were subjected to three independent replicate assays.

### Specificity

To test the cell specificity of the RGA assay, we employed the 293T cells instead of the 293T/MERS cells as target cells or Jurkat/NFAT-luc cells instead of Jurkat/NFAT-luc/FcγRIIIa as effector cells, whereas the other experimental conditions remained the same as the standard ADCC procedure. Rituximab, trastuzumab and bevacizumab were obtained from Roche, cetuximab was obtained from Merck, and these antibodies were used in the antibody specificity validation of the RGA assay.

### Stability of the cell lines

The cells were subcultured every 3 days and different passages of 293T/MERS and Jurkat/NFAT-luc/FcγRIIIa cell lines were frozen during continuous culture. The cells of different passages were thawed and assayed in the established bioassay simultaneously. The Jurkat/NFAT-luc/FcγRIIIa cells at passage number of 7, 17, 27, and 37 were selected to validate stability along with the 293T/MERS cells at passage number of 7. Similarly, the 293T/MERS cells at passage number of 7, 15, 23, 31, and 37 were tested to demonstrate their stability together with the Jurkat/NFAT-luc/FcγRIIIa at passage number of 7.

### BATDA release cytotoxicity assay

The 293T/MERS cells were cultured in solution containing DELFIA BATDA labeling reagent (Perkin Elmer) for 60 min at 37 °C. Then the labeled 293T/MERS cells were washed and counted. The labeled 293T/MERS cells and the effector cells NK92/CD16a cells were mixed at the proportion of 1:4 (2.5 × 10^4^ 293T/MERS and 1 × 10^5^ NK92/CD16a cells/well in 25 μL of dilution medium respectively), and the 50 μL mixtures were seeded into 96-well plates. Anti-MRES antibodies were serially diluted with dilution medium with a 1:4 ratio at the initial concentration of 1000 ng/mL, and 50 μL of the serial antibody diluents were added into the 96-well plates. The cultures were incubated at 37 °C for 1 h. After incubation, the plates were centrifuged and 40 μL of the supernatant from each well is transferred into a 96-well white plate containing 160 μL of Europium solution each well. The Europium-TDA fluorescence was determined by the measurement of relative time-delayed fluorescence units (RFU). Wells contained labeled 293T/MERS cells suspended in normal medium and Triton X-100 lysis buffer are for spontaneous BATDA release control and maximum release control respectively, while wells contained the cell mixtures of target and effector cells in normal medium are for background. The percentage of specific lysis was calculated using the following formula: percentage of lysis (%) = (experimental count − background count/maximum count − spontaneous count) × 100%.

### Statistical analysis

A four-parameter logistic model (Sect. 5.3 of European Pharmacopoeia) was employed to determine the 50% concentration of maximal effect (EC_50_), dose response, as well as linear range of the bioassay. The relative potency of the bioactivity was calculated by the ratio of EC_50_ value of the reference to the sample. The signal to noise ratio (SNR) was calculated using the ratio of the upper to the lower asymptotes. Data analysis and graphing were conducted using the GraphPad Prism software.

## Results

### Effect of glycosylation modification on the binding activity of anti-MERS antibody

**Figure 1:**
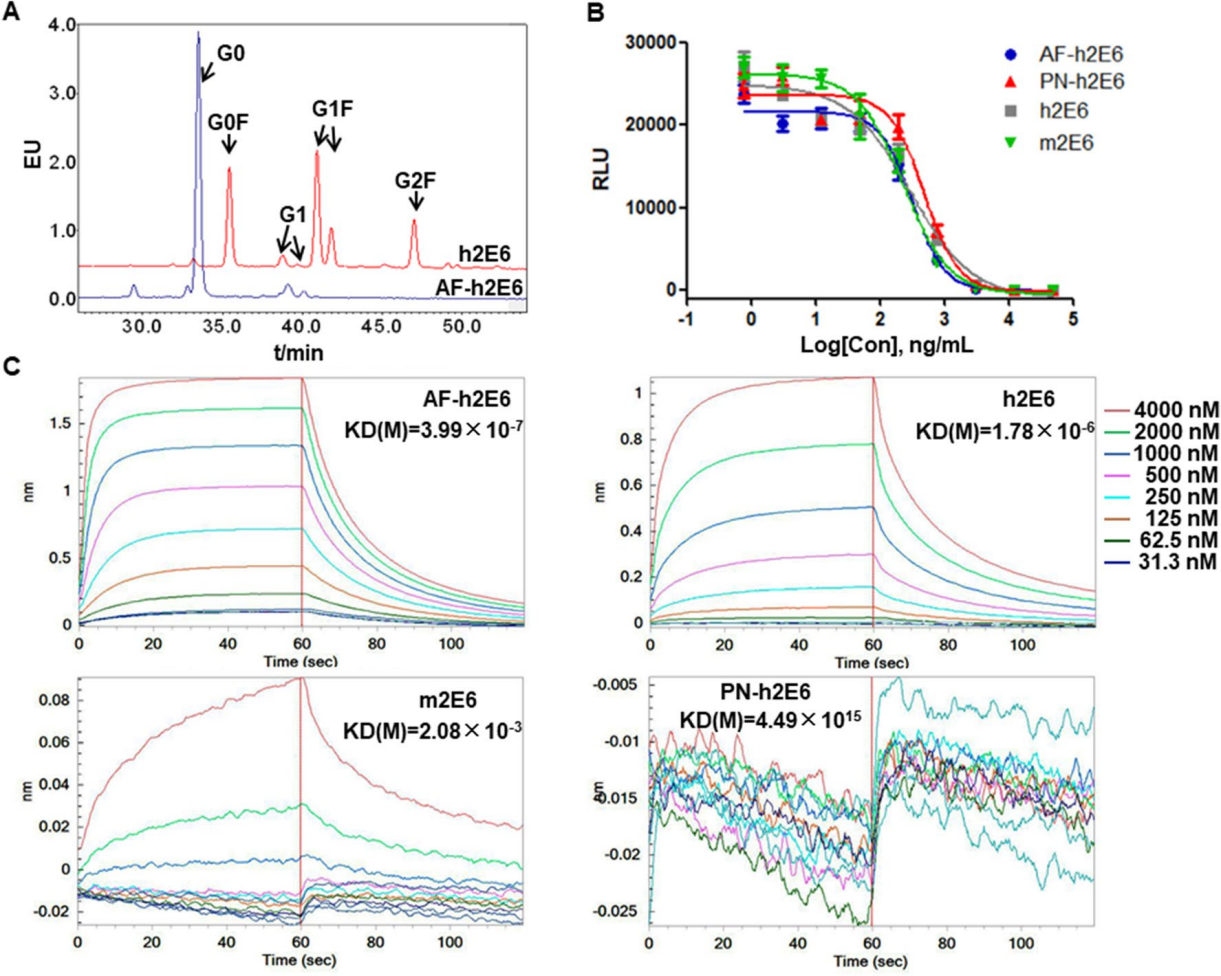
Binding activity of anti-MERS-CoV antibodies. (**A**) The glycosylation analysis of afucosylated h2E6 antibody (AF-h2E6) (blue line) and its parental control h2E6 (red line). (**B**) The pseudovirus neutralization activity of anti-MERS CoV antibodies, including m2E6 (murine 2E6), h2E6 (humanized 2E6), AF-h2E6, and PN-h2E6 (PNGase-F treated h2E6). (**C**) Kinetics of binding between anti-MERS CoV antibodies and the FcRIIIa using SPR.

The pseudovirus neutralization activity mediated by the Fab segment and the binding affinities with FcγRIIIa mediated by the Fc segment of several anti-MERS-CoV antibodies with the same antigen binding site and different Fc segments were determined. 2E6 is a neutralizing antibody against MERS-CoV targeting the receptor-binding domain of the spike protein, and were generated by mice immunized with recombinant MERS-S and subsequently selection of hybridoma cell lines. 2E6 can block virus entry in vitro with high efficacy^20^. Humanized 2E6 antibodies (h2E6) were generated by preserving the paratope residues and substituting the remaining amino acids of the murine 2E6 antibody (m2E6) with counterparts of the human immunoglobulins. Both m2E6 and h2E6 were belong to the IgG1 subtype. Afucosylated h2E6 antibody (AF-h2E6) were produced by a Chinese hamster ovary (CHO) host cell line lacking the fucose synthesis ability while the h2E6 were produced by a wildtype CHO cell line. The glycosylation of h2E6 and AF-h2E6 were analyzed and there showed nearly no glycans with core fucose in the AF-h2E6, whereas the ratio of glycans with core fucose in the h2E6 was more than 90% (G0F 30.35%, G1F 48.95%, and G2F 14.59%) (Fig. 1A). The pseudovirus neutralization assay was performed to determine the neutralization activity of the anti-MERS CoV antibodies including m2E6, h2E6, AF-h2E6, and PN-h2E6 without N-glycans. No evident differences were observed in the 50% neutralization dose (ND_50_) of these anti-MERS CoV antibodies with different sources of species and glycosylation modified Fc segments (Fig. 1B). The affinity of anti-MERS antibodies to the human FcγRIIIa(CD16a) were determined by the ForteBio Octet system, and KD values varied inversely to the affinity. The affinity of the m2E6 to human FcγRIIIa was very low [KD(M) = 2.08 × 10^–3^], and h2E6 showed certain degree of affinity to the FcγRIIIa [KD(M) = 1.78 × 10^–6^], whereas AF-h2E6, which lacked core fucose, exhibited significantly higher affinity [KD(M) = 3.99 × 10^–7^], and PN-h2E6, which was derived from AF-h2E6, did not bind to human FcγRIIIa at all [KD(M) = 4.49 × 10^15^] (Fig. 1C).

### Generation of the 293T/MERS cell line

**Figure 2:**
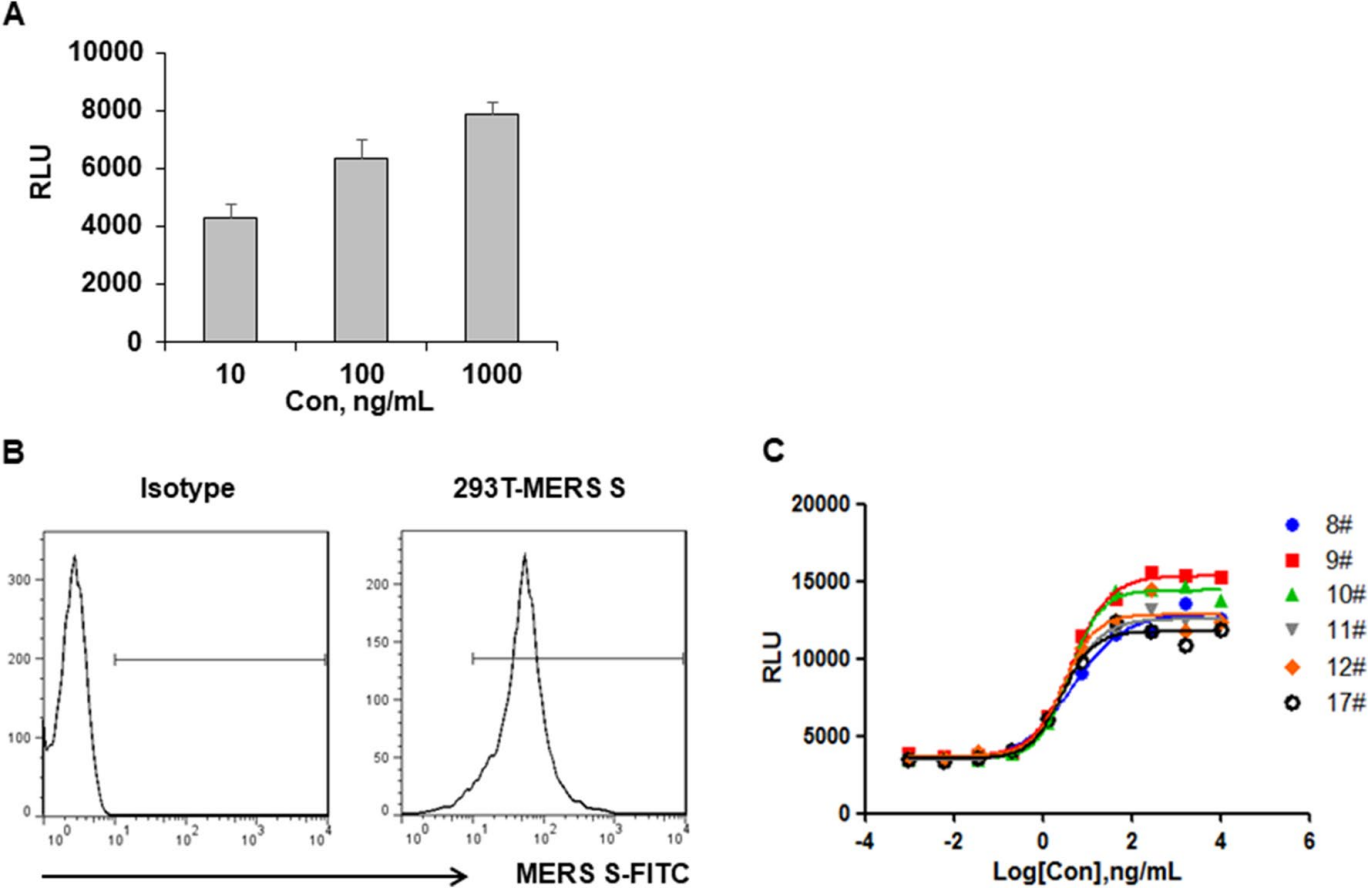
The establishment of stable 293T/MERS cell line. (**A**) 293T cells were transiently transfected with pcDNA4-MERS-S plasmid and were used as the target cell 24 h after the transfection (2.5 × 104 cells/well), while the Jurkat/NFAT-luc/FcγRIIIa cells were used as effector cells (5 × 104 cells/well). The AF-h2E6 antibodies were diluted and added to the cell mixture at a final concentration as indicated. The cultures were incubated at 37 °C with 5% CO2 for 24 h before the RLU was read. (**B**) A representative result of the antigen-positive clone screened by the flow cytometry. (**C**) 293T/MERS clone was selected by the ADCC reporter gene assay.

To verify whether the anti-MERS antibody has ADCC activity and whether our experiment designed to determine the ADCC activity is reasonable, we utilized the 293T cells which were transiently transfected with pCDNA4-MERS-S plasmid as target cells and the Jurkat/NFAT-luc/FcγRIIIa cells as effector cells to evaluate the ADCC activity of the AF-h2E6 antibody. The results showed that the relative luciferase units (RLU) was positively associated with the AF-h2E6 concentration, indicating that the AF-h2E6 has ADCC activity and can be evaluated by our RGA detection system (Fig. 2A). Then, to establish a cell line that stably expresses the MERS antigen, the 293T cells were transfected with pCDNA4-MERS-S, followed by subcloning and pressure screening. The clones showing high expression of the MERS antigen were identified by flow cytometry (Fig. 2B), and the optimized clones were subjected to the ADCC reporter gene assay subsequently (Fig. 2C). Of the six representative antigen-positive clones identified by flow cytometry, clone 9 showed the highest fold induction (FI) of luciferase under the same experimental condition in the ADCC determination assay and was named as 293T/MERS (Fig. 2C).

### Optimization of the ADCC reporter gene assay

**Figure 3:**
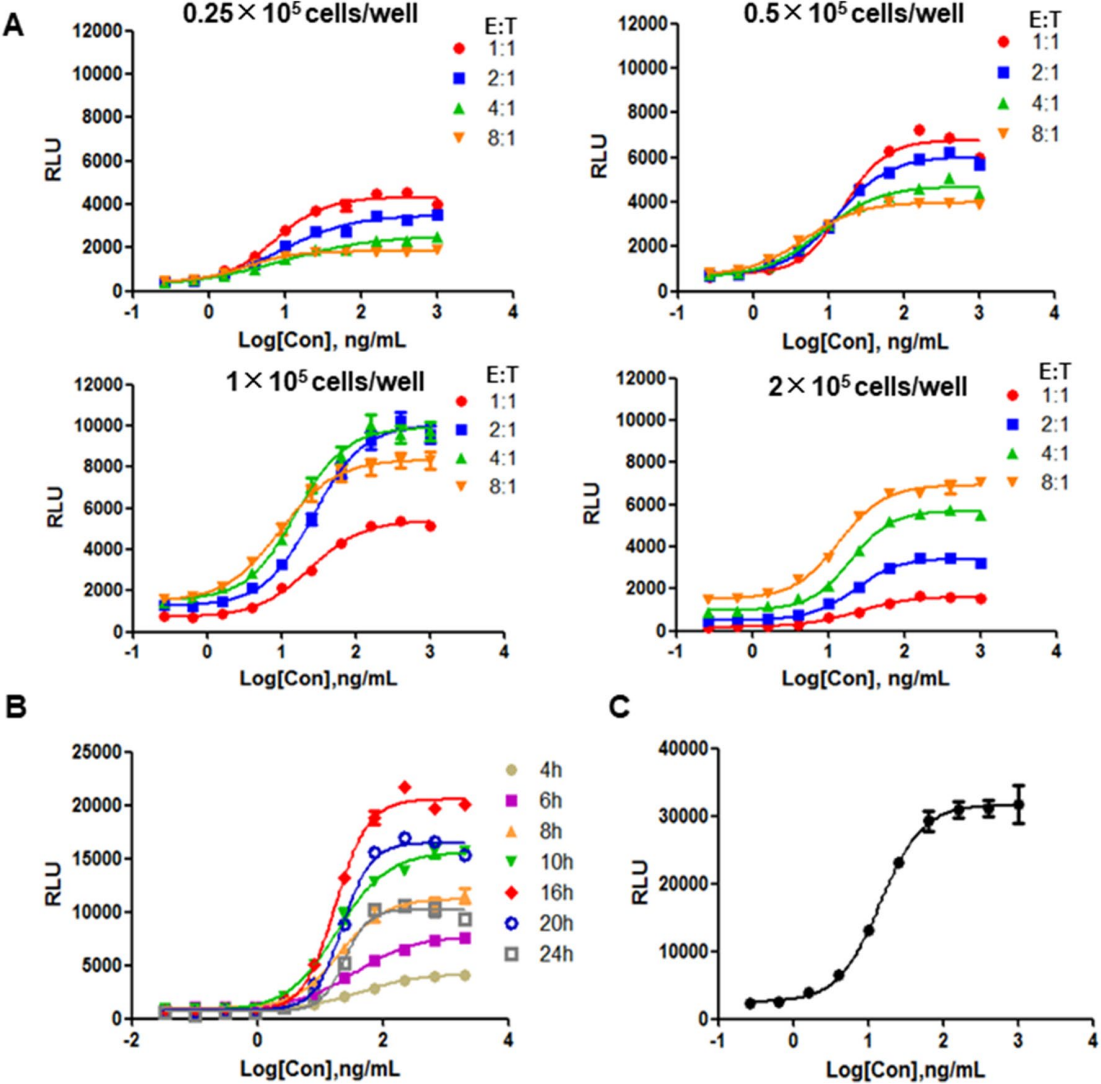
Optimization of the ADCC reporter gene assay. (**A**) Optimization of cell density. The cell density of the Jurkat/NFAT-luc/FcγRIIIa cells was adjusted to 0.25 × 105 cells/well, 0.5 × 105 cells/well, 1 × 105 cells/well, 2 × 105 cells/well, and the density of the 293T/MERS cells was correspondingly adjusted to the ratios of E:T = 1:1, 2:1, 4:1, 8:1. (**B**) Incubation time optimization. The serially diluted AF-h2E6 antibodies were co-cultured with 293T/MERS cells (2.5 × 104 cells/well) and Jurkat/NFAT-luc/FcγRIIIa cells (1 × 105 cells/well) for 4 h, 6 h, 8 h, 10 h, 16 h, 20 h, 24 h before the RLU were read. (**C**) The optimized fitting curve. The antibody concentration was initiated at a working concentration of 1 μg/mL and serially diluted in a 1:2.5 ratio with dilution medium, and the other experimental conditions were used according to the optimized results.

To establish an optimal and standardized ADCC reporter gene assay to determine the ADCC activity of the anti-MERS antibodies, we optimized the key experimental parameters of the assay. We performed a simple orthogonal experiment involving the variables of incubation time, cell density, and the ratio of effector cells to target cells, and the results showed that there was no obvious correlation between these experimental factors in this experiment (data not shown), so we optimized these factors separately by changing one factor while keeping the others constant. The cell density of the effector cells was adjusted to 0.25 × 10^5^, 0.5 × 10^5^, 1 × 10^5^, 2 × 10^5^ cells/well, and the density of the target cells were correspondingly adjusted at the ratio of the effector to target (E:T) of 1:1, 2:1, 4:1, 8:1. The dose–response curve showed good signal value and high SNR when the effector cells density was 1 × 10^5^ cells/well and the ratio of effector cells to target cells was 4:1 (Fig. 3A). For the optimization of the incubation time, the RLU values were recorded after the co-cultures incubated for 4 h, 6 h, 8 h, 10 h, 16 h, 20 h, and 24 h separately, and the highest SNR of the dose–response curve appeared about 16 h (Fig. 3B). According to the fitting curve in the previous optimization process, we optimized the antibody concentration, initiating at a working concentration of 1 μg/mL and serially diluted at a 1:2.5 ratio with dilution medium (Fig. 3C).

### Assessment of the accuracy, specificity, precision, and linearity of the ADCC reporter gene assay

**Figure 4:**
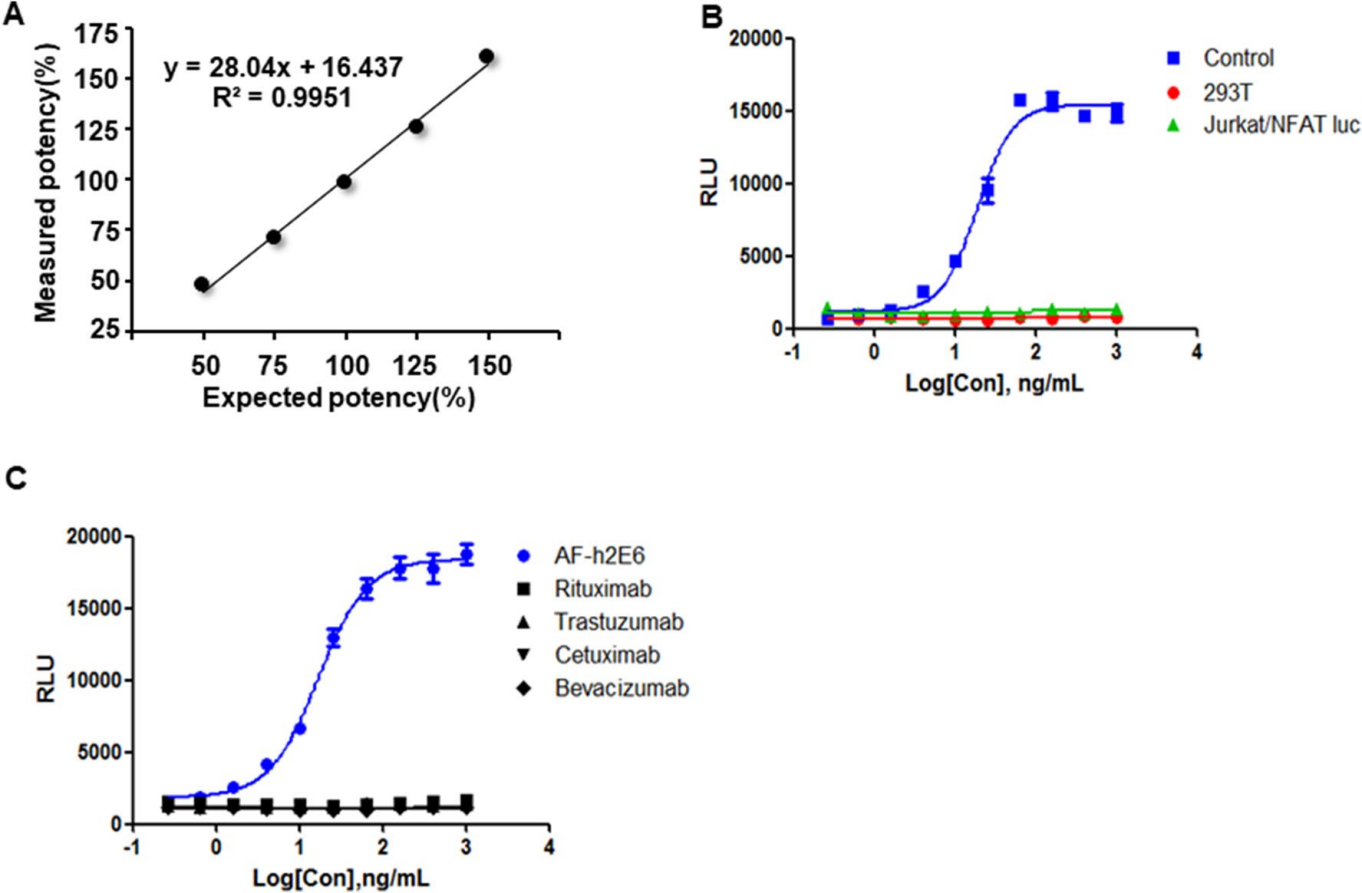
The linearity and specificity of the RGA. (**A**) Linearity of the RGA. The measured data of activity at the concentration of 50%, 75%, 100%, 125%, and 150%, relative to the established RGA, were plotted for linear regression. Each point indicates the mean of three replicates. (**B**) Cell specificity of the RGA. We replaced the 293T/MERS with the parental 293T (red line), or replaced the Jurkat/NFAT-luc/FcγRIIIa with the Jurkat/NFAT-luc (green line), the other experimental conditions were the same as the established RGA (blue line). (**C**) Specificity of the RGA to non-specific antibodies. The ADCC bioactivity of cetuximab, bevacizumab, rituximab and trastuzumab were detected in our established ADCC activity determination assay.

The ADCC reporter gene assay was validated according to the International Conference on Harmonization Q2 (ICH-Q2) guidelines^42^ by assessing the accuracy, specificity, linearity, and precision. For the precision assessment, the AF-h2E6 was diluted to three copies, one of which served as the reference, and the relative activity was the ratio of EC_50_ value of the reference to sample. Repeatability tests were performed for three times independently on different days, and the results indicated good precision (Table 1). The accuracy of an analytical procedure is an indication of agreement between the measured and expected values. The linearity of an analytical procedure indicated its ability to obtain test results that are directly proportional to the concentration of the sample analyte within a given range^43^. The AF-h2E6 anti-MERS antibody were diluted at the starting concentrations of 50%, 75%, 100%, 125%, and 150%, respectively, relative to the established antibody concentration in the RGA, using another 100% established antibody concentration as the reference, and the measured relative activity was the ratio of EC_50_ value of the reference to each sample. The relative bioactivity of each sample was determined and showed high closeness of agreement between the measured and expected biological activity, indicating excellent accuracy of the ADCC reporter gene assay (Table 2). The measured data of activity at the concentrations of 50%, 75%, 100%, 125%, and 150% were linearization analyzed and showed good linearity in the established bioassay (Fig. 4A). Specificity means that the result of the experiment is not caused by non-specific interactions between cells in the system and the assay could unequivocally evaluate the analyte of interest in the presence of other components. When we replaced the 293T/MERS with the parental 293T cells or replaced the Jurkat/NFAT-luc/FcγRIIIa with the Jurkat/NFAT-luc cells, no luminescence signals mediated by the AF-h2E6 antibody were observed (Fig. 4B). Bevacizumab (anti-VEGF mAb), even rituximab (anti-CD20 mAb), trastuzumab (anti-HER2 mAb), and cetuximab (anti-EGFR mAb) which have the ability to activate ADCC, all failed to produce a dose-dependent signal curve in our established ADCC activity determination assay for anti-MERS antibodies (Fig. 4C). These results confirmed the specificity of our assay.

**Table 1:** The repeatability of the ADCC reporter gene assay operated on different days.

	Relative activity, %		
	Day 1	Day 2	Day 3
Sample 1	105.4	103.6	108.9
Sample 2	104.2	103.7	94.3
Mean	103.3		
SD	4.86		
CV (%)	4.70

**Table 2:** The closeness of agreement between the measured and the expected biological activity.

Expected potency	50%	75%	100%	125%	150%
Mean of measured potency (%)	47.4	71.1	98.5	125.4	160.4
CV (%)	5.1	2.6	4.1	2.3	3.1
Recovery (%)	94.7	94.9	98.5	100.3	106.9

### Comparison of antibody biological activity determined by ADCC reporter assay and cytotoxicity assay

**Figure 5:**
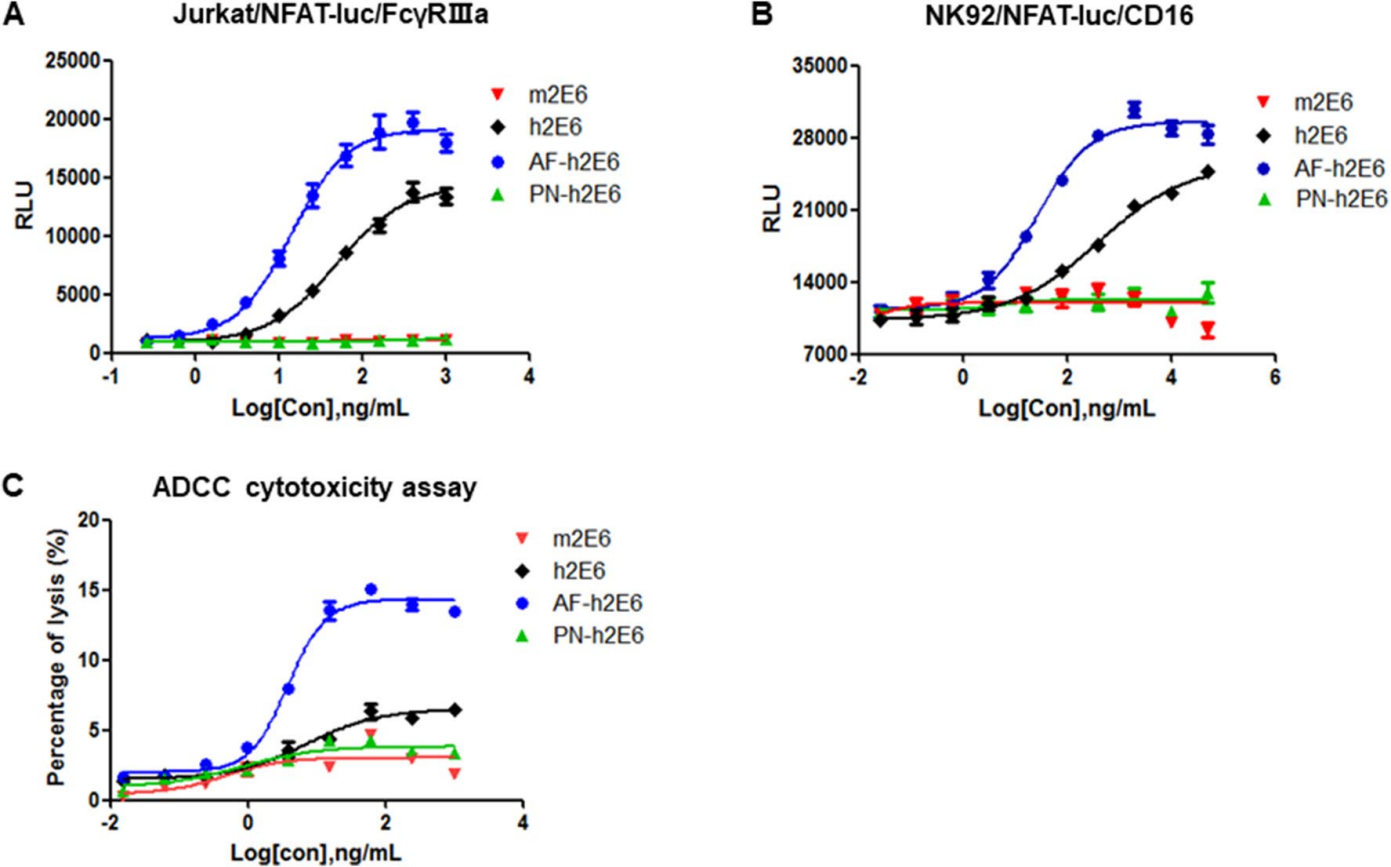
Comparison of antibody biological activity determined by ADCC reporter assay and cytotoxicity assay. The ADCC activity determination of m2E6, h2E6, AF-h2E6 and PN-h2E6 using different effector cells. (**A**) ADCC reporter assay using Jurkat/NFAT-luc/FcγRIIIa effector cells. (**B**) ADCC reporter assay using NK92/NFAT-luc/CD16 effector cells. (**C**) NK92/CD16a-based cytotoxicity assay.

ADCC is traditionally mediated by NK cells, so we evaluated the level of agreement between ADCC reporter assay using Jurkat/NFAT-luc/FcγRIIIa cells and using NK92/NFAT-luc/CD16 cells. The ADCC activity of m2E6, h2E6, AF-h2E6 and PN-h2E6 were determined. The m2E6 antibody failed to produce a dose-dependent signal curve in the ADCC detection systems based on the human Fc receptor while the h2E6 showed obvious ADCC activity. The ADCC activity enhanced significantly when the core fucose expression lacked in the AF-h2E6 antibody, whereas the ADCC activity disappeared when all of the N-glycan were removed in the PN-h2E6 (Fig. 5A,B). These results demonstrated that the ADCC activity which mediated by anti-MERS antibodies with the same Fab segment but having different glycosylated Fc segment were different. In addition, the results also proved the specificity of the determination of ADCC activity, as the luciferase activity was not caused by nonspecific cross-linking between cells or between antibodies and cells since m2E6 and PN-h2E6 failed to activate the luciferase activity in the same culture system. No significant differences were found between ADCC procedure based on the effector cell of Jurkat/NFAT-luc/FcγRIIIa (Fig. 5A) and NK92/NFAT-luc/CD16 (Fig. 5B), which demonstrated high consistency for the two assays.

Furthermore, a traditional cytotoxicity assay was performed to demonstrate that the 293T/MERS target cells were killed following incubation with anti-MERS antibodies and NK92/CD16a cells. The 293T/MERS target cells were loaded with an acetoxymethyl ester of a fluorescence enhancing ligand. After the ligand had penetrated the cell membrane, the ester bonds were hydrolyzed within the cell to form a hydrophilic ligand (TDA), which no longer passes through the membrane. The labeled target cells were incubated together with the serially diluted anti-MERS antibodies and the NK92/CD16a effector cells. When the NK92/CD16a effector cells were activated by the anti-MERS antibodies bound on the target cells, the TDA-labeled target cells were lysed and the TDA was released in the cell culture supernatant. The subsequently measured fluorescence signal, emitted by the chelate between the added europium and the released TDA, was correlated to the number of lysed target cells. The results showed that the death of target cells mediated by ADCC was similar with the RLU signal in the reporter assay (Fig. 5C). These results demonstrate that the luciferase activity detected in the reporter assay can be an indirect measure of ADCC.

### Stability of the 293T/MERS and Jurkat/NFAT-luc/FcγRIIIa cell lines

**Figure 6:**
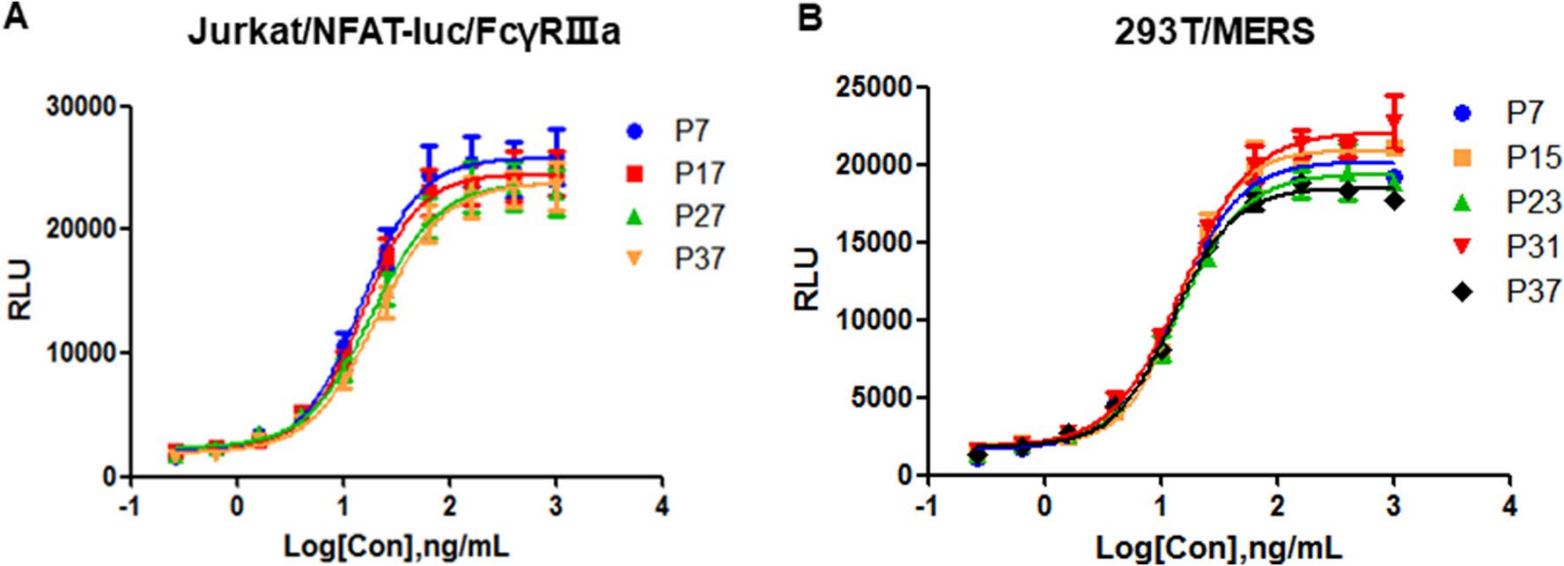
Stability of the 293T/MERS and Jurkat/NFAT-luc/FcγRIIIa cell lines. The ADCC activity of h2E6 was determined using different target cells or effector cells of different passage number. (**A**) The Jurkat/NFAT-luc/FcγRIIIa cells at passage number of 7, 17, 27, and 37 were selected to validate stability along with the 293T/MERS cells at passage number of 7. (**B**) The 293T/MERS cells at passage number of 7, 15, 23, 31, and 37 were tested to demonstrate their stability together with the Jurkat/NFAT-luc/FcγRIIIa at passage number of 7.

The stability of the transgenic cell line is crucial to the robustness and consistence of the reporter gene assay. The 293T/MERS and Jurkat/NFAT-luc/FcγRIIIa cell lines of different passage number were frozen during continuous cultures and the stability of the cell lines were tested in the established bioassay. When verifying the stability of different passage number of one cell line, we keep the other cell line constant. The results showed that when the passage number of the two cell lines was between 7 and 37, there were no significant differences of the overall fluorescence intensity and the signal–noise ratio of the dose–response curves (Fig. 6A and 6B), demonstrating excellent stability of the 293T/MERS and Jurkat/NFAT-luc/FcγRIIIa cell lines and robustness and consistency of the established bioassay.

## Discussion

Bioactivity is a key quality attribute of therapeutic antibodies. In recent years, the therapeutic antibodies have been rapidly developed, raising the high requirement for bioactivity determination assay. ADCC activity serves as a critical mechanism of action of many therapeutic antibodies, including antiviral antibodies^31–36^. The effector cells used in the classic ADCC assay are primary human peripheral blood mononuclear cells (PBMCs) and the subset natural killer (NK) cells that are isolated from human blood. The target cells are pre-loaded with radioactive tracer such as radioactive chromium (51Cr)^44^ or fluorescent dyes such as calcein-AM^45,46^, and the release of these substances are detected to determine the cytotoxicity. In addition, ADCC activity can also be measured by detection of cell death and proliferation such as the measurement of released LDH^47^. All of these assays have poor reproducibility and low sensitivity, due to the inherent variability of the primary effector cells and the high levels of spontaneous release independent on the antibody-dependent killing of target cells. These highly variable traditional methods are thus not suitable for routine quantitative measurement of ADCC activity of therapeutic antibodies^48^. Parekh et al. developed a reporter gene assay as a surrogate ADCC assay for the bioactivity determination of anti-CD20 antibody, in a model system with CD20^+^ B cell line WIL2-S cells and a Jurkat-based cell line stably expressing human FcγRIIIa and an NFAT response element regulating a luciferase reporter^41^. Cheng et al. evaluated several other antibodies with appropriate target cells by a similar reporter-based surrogate ADCC assay^49^. The ADCC reporter assay showed improved precision and overall consistence compared to the classic ADCC assay.

In our previous work, we have established several novel bioactivity determination methods for different kinds of antibodies based on reporter gene assays, and these methods have obvious advantages compared to the classic bioactivity determination methods if there ever were^43,50,51^. The documented reporter-based surrogate ADCC assay were all designed for antitumor antibodies targeted to hematopoietic or solid tumor cells^41,49^, and there have been no reports about surrogate ADCC assay for antivirus antibodies. Different from methods for antitumor antibodies, there are no natural target cells for antiviral antibodies, so it would be of great value to design an ADCC reporter gene assay for antiviral antibody.

The target cells used in the ADCC activity determination assay for the antiviral antibodies, unlike the ready-to-use tumor cell lines used for antitumor antibodies, were prepared by transient infection of the susceptible cells with live viruses or pseudoviruses^52–54^, so the preparation procedure is complex, and the target cells are unstable and highly variable. In this study, we generated a 293T/MERS cell line stably expressing the MERS-S as the target cell in the ADCC assay (Fig. 2). The effector cells used in the reporter assay are engineered Jurkat cells expressing the human Fc receptor FcγRIIIa (CD16a) and a luciferase reporter gene driven by NFAT response elements. Jurkat cells are CD4^+^ T cells, and do not have cytotoxic capability as effector cells. We selected the engineered Jurkat cells rather than NK cells, because Jurkat cells share the same ability to activate the appropriate pathways for cell activation after the crosslinking of FcγRIIIa as that of NK cells^55,56^ and Jurkat cells are much more simple to handle than NK cell line. The ADCC reporter assay using Jurkat/NFAT-luc/FcγRIIIa is more suitable for release control, batch-to batch consistency and stability tests for mAbs with ADCC activity where accuracy and precision are important, and engineered Jurkat cells are commonly employed in the surrogate ADCC measurement of mAbs^41,49^ and cytotoxicity measurement of CD3 based bispecific antibodies (BiTEs)^57^ in industry.

When the anti-MERS antibody recognize the MERS-S antigen on the surface of 293T/MERS cells, the Fc segment of the antibody bind to the FcγRIIIa on the Jurkat/NFAT-luc/FcγRIIIa cells and initiate the downstream signaling pathways, resulting in the expression of the luciferase reporter gene driven by NFAT elements. The luciferase expression was positively associated with the concentration of anti-MERS antibodies, and the bioactivity of anti-MERS antibodies can be determined by the measurement of RLUs. For the application of this new ADCC reporter assay in the research and development of vaccines and antibodies, we optimized the experimental conditions of this method, and obtained a typical dose-dependent curve with suitable SNR and uniform distributed data points (Fig. 3).

In addition, the precision, accuracy, linearity, and specificity of the analytical method were evaluated according to the ICH-Q2 analytical method guidelines. The small value of coefficient of variation (CV) demonstrated the excellent precision (Table 1). The high consistency between the measured and the expected biological activity demonstrated good accuracy (Table 2). Besides, the method showed good linearity in the concentration ranges of 50%–150% (Fig. 4A). By replacing the target cells or the effector cells with the corresponding parental cells, no non-specific signals in the cells caused by stimulation of the anti-MERS antibody were observed (Fig. 4B). Furthermore, only anti-MERS antibodies but not non-specific antibodies could induce luciferase expression in the ADCC assay (Fig. 4C), proving the high specificity of this ADCC assay. The ADCC assay also showed sufficient sensitivity in detecting the different bioactivity of different anti-MERS antibodies derived from different species or modified with different glycans (Fig. 5A). Cell passage number may affect the stability of transgenic reporter gene cells, and our results proved the excellent stability of the 293T/MERS and Jurkat/NFAT-luc/FcγRIIIa cell lines (Fig. 6).

NK cells are the key mediators of ADCC activity. The bioactivity of anti-MERS antibodies were determined in two ADCC reporter assays using Jurkat/NFAT-luc/FcγRIIIa effector cells (Fig. 5A) and NK92/NFAT-luc/CD16 effector cells (Fig. 5B) separately, and the results showed highly consistency. In addition, a traditional cytotoxicity assay mediated by NK92/CD16a effector cells was performed to prove that the results were concordance with those of the established RGA, and the similar correlation of antibody glycosylation with ADCC biological activity of different anti-MERS antibodies demonstrated that the luciferase activity driven by FcγRIIIa-NFAT signaling pathway in the effector cells can be a convincing indication of ADCC activity (Fig. 5C). The ADCC bioactivity of the anti-MERS antibodies is closely related to the affinity of Fc segment of antibody to the Fc receptor FcγRIIIa (Fig. 1C), whereas differ from the neutralization activity mediated by the Fab segment of these antibodies (Fig. 1B). Consistent with published reports^58,59^, our results showed that the afucosylated anti-MERS antibody AF-h2E6 exhibits higher binding affinity to CD16a (Fig. 1C) and increased ADCC potency than their fucosylated counterparts h2E6 (Fig. 5).

In the current study, we developed a robust reporter-based ADCC assay for the determination of bioactivity of anti-MERS antibodies targeting MERS spike protein for the first time. The ADCC assay will be a useful tool in the antibody characterization and lot release of therapeutic antibodies. Furthermore, our study provides a method for improving the ADCC bioactivity of anti-MERS antibodies by N-glycosylation modification of the Fc segment, which has been reported in other antiviral antibodies such as Ebola^60^, while there are no reports on the ADCC activity of anti-MERS antibodies nor corresponding ADCC determination methods to date. As for now, this ADCC activity determination method is validated for mAbs targeting MERS spike protein but not for the corresponding spike-specific antibodies in human serum. We believe that the MERS-CoV-specific ADCC assay is highly applicable in the screening, characterization, release control, and stability study of MERS-CoV neutralizing antibodies which are mostly targeting RBD region of spike protein, and may provide good technical rational for other anti-coronaviruses research.
